# Supplementary material for: Raman developmental markers in root cell walls are associated with lodging tendency in tef
Source: Planta. 2024 Jan 31;259(3):54. doi: 10.1007/s00425-023-04298-7 (PMC10830713; doi:10.1007/s00425-023-04298-7)
Supplement: Supplementary file 1 — Supplementary file1 (DOCX 1707 kb) [file 425_2023_4298_MOESM1_ESM.docx]

**Raman developmental markers in root cell walls are associated with lodging tendency in tef**

*Sabrina Diehn^1^, Noa Kirby^1^, Shiran Ben-Zeev^1^, Muluken Demelie Alemu^1,2^, Yehoshua Saranga^1^, Rivka Elbaum^1^*

*^1^The Robert H. Smith Institute of Plant Sciences and Genetics in Agriculture, The Hebrew University of Jerusalem, Rehovot 7610001, Israel*

*^2^Ethiopian Institute of Agricultural Research, Ethiopia*

**Supporting table and figures**

**Table S1:** Days to flowering (DTF) and lodging indices at week 8, 9, and 10 (LI8, LI9, and LI10, respectively) of the four tef genotypes (RTC-157, RTC-275, RTC-392, RTC-400) in a field experiment, Shiller Farm 2021. Data are means of two six plots (2 irrigation regimes X 3 replicates). Different letters (a,b,c) indicate significant difference according to Tukey HSD test (*P*<0.05), *, ** and *** indicate significant F ratios at *P*<0.05, 0.01 and 0.001, respectively. Data adapted from Demelie Alemu and Ben-Zeev (pers. communication, Hebrew University of Jerusalem, 2023).

| Effect of genotypes | | DTF | LI8 | LI9 | LI10 |
| --- | --- | --- | --- | --- | --- |
| RTC-157 |  | 81.2 a | 37 b | 285 b | 387 c |
| RTC-275 |  | 54.2 b | 183 b | 417 b | 550 b |
| RTC-392 |  | 43.7 c | 117 b | 450 b | 667 ab |
| RTC-400 |  | 57.2 b | 450 a | 833 a | 783 a |
|  |  |  |  |  |  |
| Source | DF | F Ratio | F Ratio | F Ratio | F Ratio |
| ENV | 1 | 126 | 2.69 | 21.97 *** | 122.47 *** |
| Geno | 3 | 4519.1 *** | 10.72*** | 9.59 ** | 26.00 *** |
| ENV*Geno | 3 | 353.8 * | 1.45 | 1.82 | 13.8 *** |
| Block | 2 | 35.6 | 2.21 | 0.24 | 0.81 |


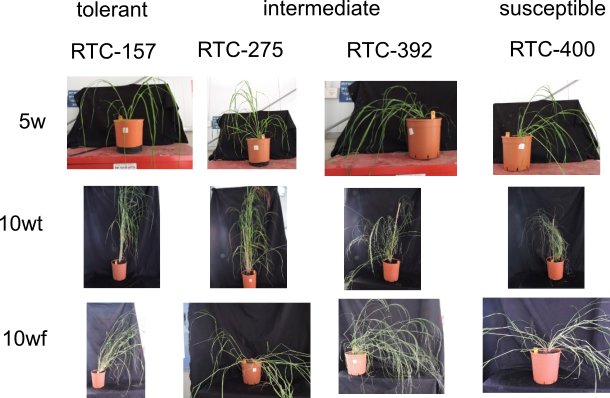


**Fig. S1** Photographs of plants that were used in this study, including (from left to right) RTC-157, RTC-275, RTC-392, and RTC-400 genotypes of plants of (top to bottom) 5-week-old (5w), supported 10-week-old (10wt), and non-supported 10-week-old (10wf).


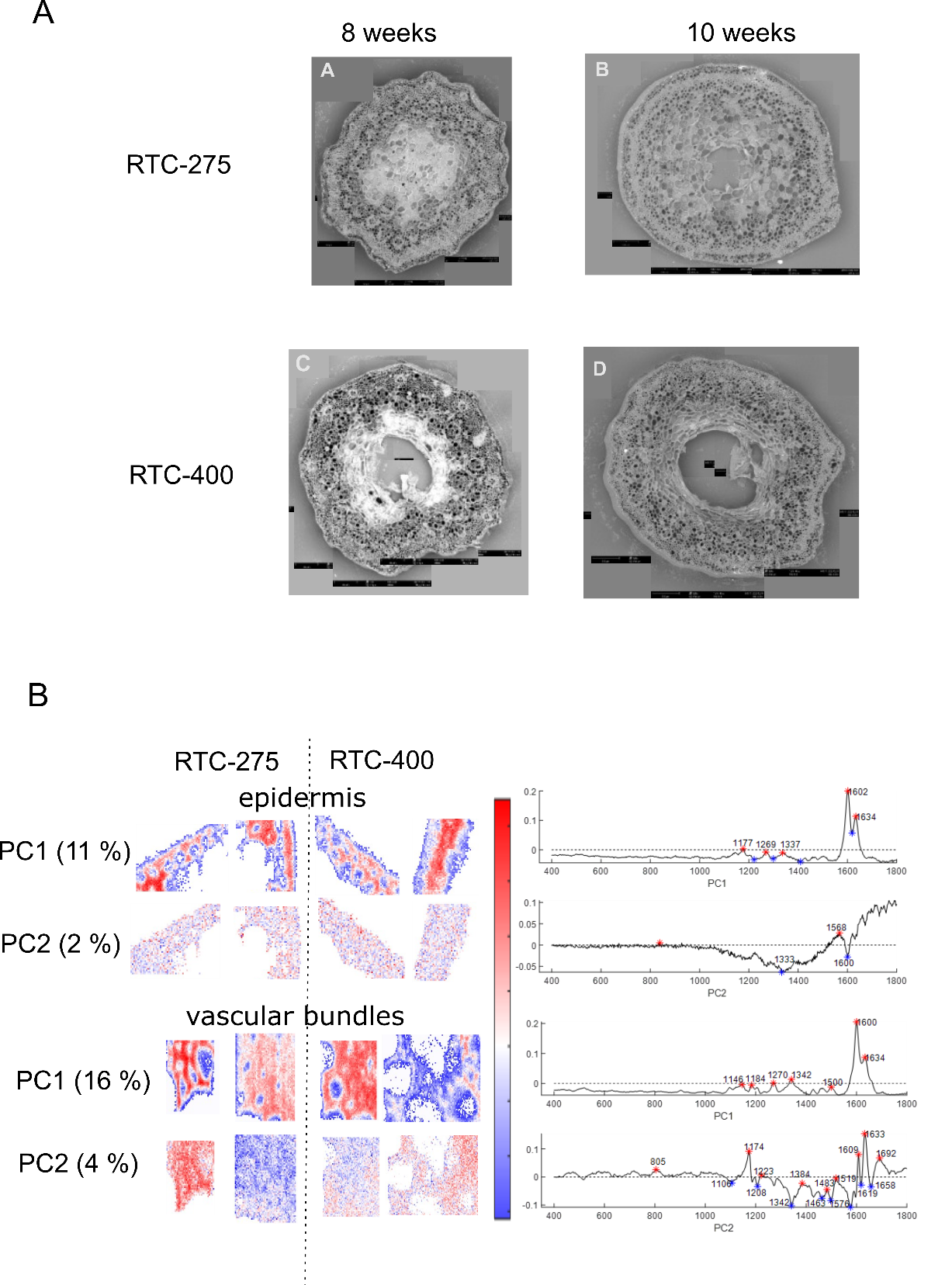


**Fig. S2** **A.** SEM mosaic images of cross sections from the first internodes of the two genotypes RTC-375 (intermediate lodging tolerant) and RTC-400 (lodging susceptible) at 8 weeks and 10 weeks after emergence. **B.** PCA images of PC1 and PC2 of internode cross sections focusing on epidermis and vascular bundles. These preliminary observations indicated a faster development of a hollow stem in the lodging susceptible genotype. Nonetheless, we could not detect consistent chemical variation linked to lodging tendencies by Raman microspectroscopy.


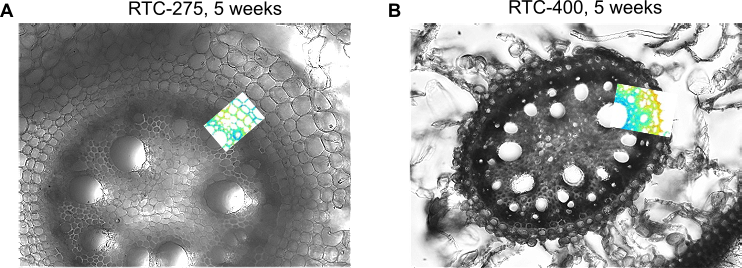


**Fig. S3** Crown root cross sections from a line with intermediate (**A**, RTC-275) and with high (**B**, RTC-400) lodging tendencies of five weeks old (5w) tef plants. The images present an overlay of bright-field images and Raman intensity maps at 1144 cm^-1^, which is tentatively associated with suberin. The endodermis inner tangential wall exhibits high suberin levels, mostly in RTC-400.


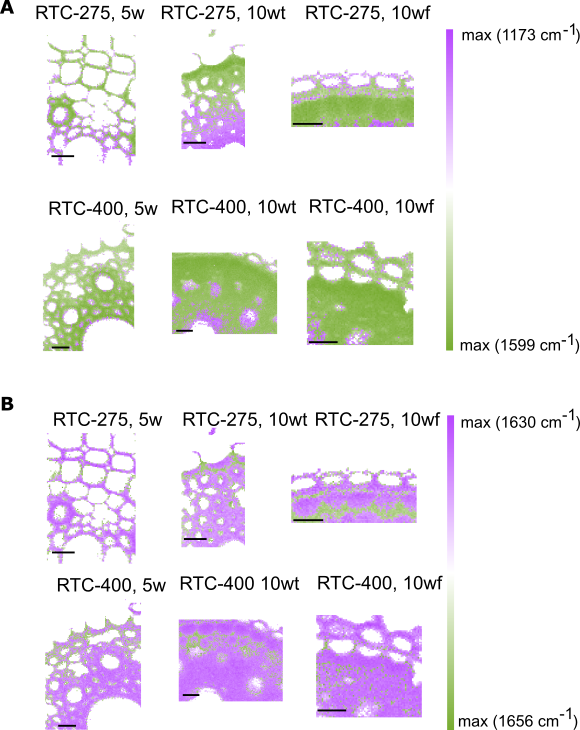


**Fig. S4** Overlays of the intensity maps of (**A**) 1173 and 1599 cm^-1^, as well as of (**B**) 1630 and 1656 cm^-1^ from 5 weeks (5w), 10 weeks trellised (10wt) and 10 weeks non-trellised (10wf) plants from the two genotypes RTC-275 and RTC-400. One can appreciate that on a cell level, the bands at 1173 and 1599 cm^-1^ and at 1630 and 1656 cm^-1^ complement each other. All scale bars represent 20 µm.
